# Potential for evolution of complex defense strategies in a multi-scale model of virus-host coevolution

**DOI:** 10.1186/s12862-016-0804-z

**Published:** 2016-10-26

**Authors:** Jeewoen Shin, Thomas MacCarthy

**Affiliations:** 1 Department of Applied Mathematics and Statistics, Stony Brook University, Stony Brook, NY USA; 2 Laufer Center for Physical and Quantitative Biology, Stony Brook University, Stony Brook, NY USA

**Keywords:** Virus entry, Host cell surface receptor, Gene regulatory network

## Abstract

**Background:**

Host resistance and viral pathogenicity are determined by molecular interactions that are part of the evolutionary arms race between viruses and their hosts. Viruses are obligate intracellular parasites and entry to the host cell is the first step of any virus infection. Commonly, viruses enter host cells by binding cell surface receptors. We adopt a computational modeling approach to study the evolution of the first infection step, where we consider two possible levels of resistance mechanism: at the level of the binding interaction between the host receptor and a virus binding protein, and at the level of receptor protein expression where we use a standard gene regulatory network model. At the population level we adopted the Susceptible-Infected-Susceptible (SIS) model. We used our multi-scale model to understand what conditions might determine the balance between use of resistance mechanisms at the two different levels.

**Results:**

We explored a range of different conditions (model parameters) that affect host evolutionary dynamics and, in particular, the balance between the use of different resistance mechanisms. These conditions include the complexity of the receptor binding protein-protein interaction, selection pressure on the host population (pathogenicity), and the number of expressed cell-surface receptors. In particular, we found that as the receptor binding complexity (understood as the number of amino acids involved in the interaction between the virus entry protein and the host receptor) increases, viruses tend to become specialists and target one specific receptor. At the same time, on the host side, the potential for resistance shifts from the changes at the level of receptor binding (protein-protein) interaction towards changes at the level of gene regulation, suggesting a mechanism for increased biological complexity.

**Conclusions:**

Host resistance and viral pathogenicity depend on quite different evolutionary conditions. Viruses may evolve cell entry strategies that use small receptor binding regions, represented by low complexity binding in our model. Our modeling results suggest that if the virus adopts a strategy based on binding to low complexity sites on the host receptor, the host will select a defense strategy at the protein (receptor) level, rather than at the level of the regulatory network - a virus-host strategy that appears to have been selected most often in nature.

**Electronic supplementary material:**

The online version of this article (doi:10.1186/s12862-016-0804-z) contains supplementary material, which is available to authorized users.

## Background

Viruses and their hosts engage in evolutionary arms races in the form of continuous molecular level changes that determine the mechanisms of infection and defense [1–4]. The evolutionary dynamics are determined in large part by host susceptibility and viral pathogenicity and ultimately depend on molecular interactions between genes and their products [5–7]. These relentless evolutionary arms races drive genetic diversity in both host and pathogen [2, 8, 9]. More generally, host-pathogen interactions have been proposed as a major factor in the evolution of biological complexity [10–13].

If we consider humans and other higher organisms as potential hosts, they will usually evolve at much slower rates than the viruses that infect them [14]. At the same time these hosts are highly complex organism and will usually have far greater resources in terms of potential defense mechanisms and, more generally, in terms of genetic information to deal with the viral infections. Viral entry will commonly involve binding interactions with receptors on the host cell surface [15, 16]. Most host cells will have a large number of cell surface receptors, many of which are involved in essential functions such as detection of signaling molecules (e.g. hormones) or nutrients, but which can be usurped by viruses as cell entrance mechanisms [17, 18]. Functional redundancy among receptors is common. For example, nectins are cell entry receptors of Herpes simplex virus (HSV) and are involved in cell adhesion. Functional redundancy within the nectin family and also other cellular adhesion proteins can compensate for particular nectins [19]. Also, in humans there are 19 known chemokine receptors which activate the same chemokine signaling pathway but some of these have highly specific receptor binding ligands whereas others may bind multiple ligands [20]. Interestingly, some viruses produce mimics of chemokine receptor binding ligands, or may encode their own chemokines and chemokine receptors [21]. For example, CCR5 and CXCR4 act as co-receptors for HIV-1 entry [22], and the Respiratory Syncytial Virus (RSV) produces its own version of the chemokine CXC3 which binds to the host receptor CX3CRI, thus facilitating RSV infection [23].

While there are multiple mechanisms of infection and resistance across many levels, virus entry into the host cell is the first and essential step that must succeed for a viral infection to proceed [15, 16]. Thus, preventing virus entry has been often been the preferred strategy for therapeutic development [15, 24, 25]. On evolutionary timescales, hosts can evade receptor-mediated viral entry in several ways including amino acid changes at the binding sites to inhibit protein interactions, or by regulation of receptor gene expression. Several previous studies have provided evidence of evolutionary arms races at the level of virus-receptor protein interactions. For example, Transferrin Receptor-1 (TfR1) is a key regulator of iron uptake in mammalian cells and is up-regulated when intracellular iron concentrations are low [17]. However, TfR1 is also used for cell entry by viruses such as the Mouse mammary tumor virus (MMTV) and the Machupo virus. Clear evidence of positive selection has been found both on the binding sites of TfR1 for MMTV and Machupo virus and on the corresponding sites in the virus proteins that bind these [26–29]. Mutations at these residues affect receptor-binding interactions and change virulence and host susceptibility, suggesting an ongoing evolutionary arms race. Regulation of host cell surface receptors can also be an effective defense strategy against virus entry [24, 25, 30, 31]. For example, there appears to be significant variation across human bladder cells for mRNA and protein expression levels of the Coxsackie and Adenovirus Receptor (CAR) gene, another virus-targeted receptor. Thus, the T24 bladder cell line has very low CAR expression and is resistant to virus entry, whereas RT4 cells have high CAR expression level and are highly susceptible to infection [32]. Thus, regulatory changes affecting cell surface receptor levels are related to susceptibility to viral infection. Clearly, however, there may be a tradeoff between reduced receptor expression and the fitness gained by reduced infectivity, which may explain why there are many more published examples of virus-receptor coevolution than for receptor expression evolution (virus-receptor coevolution is also easier to study, so ascertainment bias may also be a factor).

Thus, hosts may adopt different resistance mechanisms at different system levels, e.g., receptor binding vs regulation. However, little previous research has focused on how these different levels of defense mechanisms may evolve in the context of host-pathogen co-evolution. Computational models such as the gene regulatory network evolution model (also known as the Wagner model), that combine a complex genotype-phenotype mapping (describing a gene regulatory network) with evolutionary dynamics have previously been used to address a range of questions concerned with evolution of biological complexity [33, 34]. In previous studies, the gene regulatory network evolution model has been extended to account for different system levels, including transcription factor (TF)-DNA binding interactions [35] and protein-protein interactions (PPI) [36] at the microscopic level, or between two different populations [10] at the macroscopic level. These previous studies [10, 36] showed how robustness and evolvability can evolve to be distributed across different system levels, depending on the model conditions. Here, we integrate protein-protein interactions (virus-receptor binding) and gene regulatory networks (which control receptor expression) in the context of an evolutionary model that represents both host and pathogen populations.

Viral proteins commonly evolve to mimic receptor binding sites in order to enter host cells through cell surface receptors [21, 26–29]. We introduce a model where the host receptor and the corresponding viral protein are represented as linear sequences and binding is quantified by a similarity score, under the assumption that a close match corresponds to better binding and a higher probability of viral entry. Hosts can evolve to block viral entry either via binding site mismatches or by regulatory changes in receptor protein expression. We further investigate how hosts evolve resistance to different types of viruses: specialists (that target a single receptor) vs generalists (that target many receptors). We consider how the balance between receptor binding and regulation evolves in the context of host-pathogen co-evolution and the need for virus to enter the host cell and the host to block virus entry. More generally, we consider what evolutionary conditions might drive a shift from protein-protein interaction towards gene regulation, and thus increased biological complexity, a key question in the field of evolutionary biology [37, 38]. Furthermore, because we specifically consider host-pathogen coevolution, our study begins to address how complex immune systems may have evolved.

## Methods

### Host-virus coevolution model

The individual gene regulatory network (GRN) structure and gene expression dynamics largely follows the original gene regulatory network evolution model [39–41], with 3 primary differences: (i) host individuals are represented by a GRN together with a set of receptor binding site sequences, (ii) populations follow the dynamics of an SIS model, and (iii) the selection pressure on hosts is given by differential survival probability for the offspring of susceptible vs infected parents and by the rate of disease-related death for infected hosts as selection on the hosts arises from the advantage that resistant offspring have over non-resistant offspring (Additional file 1).

A host GRN is represented as a matrix (*W*) of size *N* × *N*_*TF*_ where *N* is the total number of genes, which includes receptor genes (*N*_*R*_) and the transcription factor genes (*N*_*TF*_) that regulate them. Each element, *w*_*ij*_ indicates a regulation of the gene *i* by a gene product of the gene *j*, and can represent activation (*w*_*ij*_ > 0), inhibition (*w*_*ij*_ < 0), or no regulation (*w*_*ij*_ = 0). The network density (*c*) is a parameter of the model and is defined as the fraction of nonzero *w*_*ij*_ elements in the matrix *W*. A founder host individual has a randomly assigned *W* with a given network density *c* and with each nonzero *w*_*ij*_ element drawn from a Normal distribution, *N*(0, 1). Each row *i* of the matrix *W* represents the *cis*-regulatory elements of the *i* ^*th*^ genes. The GRN is composed of two sub-networks. The first sub-network, from the 1st row to the *N*_*TF*_^*th*^ row corresponds to the transcription factor (TF) genes and the second sub-network, from the *N*_*TF* + 1_^*th*^ row to the last *N* ^*th*^ row corresponds to the *N*_*R*_ receptor genes. The expression levels of the *N* genes at time *t* are represented as a vector *S*(*t*) where the *i* ^*th*^ element *S*_*i*_(*t*) corresponds to the gene expression of *i* ^*th*^ gene. A sub-vector of *S*(*t*) of TF genes (*S*_1_(*t*) ~ *S*_*TF*_(*t*)) is called *S* ^*TF*^(*t*), and a sub-vector of *S*(*t*) of receptor genes (*S*_*TF* + 1_(*t*) ~ *S*_*N*_(*t*)) is called *S* ^*R*^(*t*). Initial gene expression *S*(0) is set as a random binary vector where 0 corresponds to no gene expression and 1 is for full gene expression. Gene expression levels are updated according to the equation *S*(*t* + 1) = *Sig*(*W* ⋅ *S* ^*TF*^(*t*)), where $Sig(x)=\frac{1}{1+{e}^{- ax}}\kern0.5em \left(a=100\right)$ is a sigmoid function which maps values to gene expression levels in the range (0, 1). Here, 0.5 corresponds to basal (unregulated) gene expression. When the gene expression dynamics *S*(*t*) reach steady state [34] we simplify gene expression to binary form by applying the function $$ \varphi (x)=\left\{\begin{array}{c}\hfill 0,\kern0.5em x\le 0.5\hfill \\ {}\hfill 1,\kern0.5em x>0.5\hfill \end{array}\right. $$, thus defining the phenotype *Ŝ*.

In the model, we assume there is some degree of functional redundancy for cell surface receptors. Among the total number (*N*_*R*_) of receptors which can be expressed on the cell surface, a subset (*N*_*ER*_) is required to satisfy the minimum demand for normal host functions. Here we tested *N*_*ER*_ = 1 or 3 among *N*_*R*_ = 5 receptors. For example, *N*_*ER*_ = 1 indicates that expression of any single receptor is sufficient for host function and any receptor can substitute for any other. At the other extreme, if *N*_*ER*_ = 5 then all receptors must be expressed and there is no functional redundancy. There are multiple examples showing that different receptors on a host cell can be targeted for virus entry and also that a single host receptor can be targeted by different viruses [15, 16]. Hence, offspring individuals whose phenotypes have fewer expressed receptor genes than *N*_*ER*_ (1 ≤ *N*_*ER*_ ≤ *N*_*R*_) are assigned zero fitness since we assume that this is the minimum required for normal host cell functions. The expressed receptor genes produce cell surface receptor proteins that can be targeted by viruses for entry. Each receptor protein is represented as a binary vector of length *L*, where 0 indicates a polar amino acid and 1 indicates a hydrophobic amino acid. To represent different receptors on the host cell surface, an amino acid sequence is assigned to each receptor protein independently (we avoided having a homogeneous set of initial host receptor proteins as we found this caused population decay due to extremely beneficial conditions for the virus infection). While a host individual is represented with a GRN together with a set of receptor proteins, each virus is represented only by the protein used to enter host cells, represented also as a binary vector of length *L*.

The initial host population is created in the form of *M* clones of a founder individual possessing a randomly assigned matrix *W* and set of receptor amino acid sequences. The host population iterates through cycles of reproduction, mutation and stabilizing selection (similarity to the phenotype of the founder) for 500 time steps in order to generate genetic diversity within the population before the viruses are introduced [34]. Under asexual reproduction each offspring individual is cloned from a random parent, whereas under sexual reproduction each offspring has two random parents and inherits genes (protein sequences and *cis*-regulatory regions) from either parent randomly assuming free recombination among the genes. Since each row represents the *cis*-regulatory region of each gene, sexual reproduction involves copying each row of *W* from either of the parents for all *N* genes. GRN mutations change regulatory interactions between genes. As used previously [41], we allow interaction addition (*w*_*ij*_ = 0 → *w*_*ij*_ ≠ 0), deletion (*w*_*ij*_ ≠ 0 → *w*_*ij*_ = 0), and modification (*w*_*ij*_ = *w*_*ij*_^′^ ≠ 0 → *w*_*ij*_ = *w*_ij_^*^ ≠ *w*_*ij*_^′^, 0). The mutation frequency per matrix *W* is *μ* including addition (*ρ*), deletion (*ϕ*) and modification (*δ*). *ρ* and *ϕ* are set to satisfy $\varDelta c=c\left(t+1\right)-c(t)=\frac{\mu }{N\cdot {N}_{TF}}\cdot \left\{\rho \left(1-c(t)\right)-\phi c(t)\right\}=0$ so that the network density (*c*) remains close to that of the founder. Before contact with viruses, the host population size is fixed and hosts evolve under stabilizing selection to be close to the founder’s gene expression phenotype and expressed receptor amino acid sequences. Under stabilizing selection, a host whose phenotype has more than one gene expression difference is not able to survive. Protein mutations involve switching between 0 (polar) and 1 (hydrophobic), where the mutation probability is *μ*_*hp*_ per set of receptors. Also for the receptor similarity, we measured a fitness value $f={e}^{-\frac{D}{\sigma }}$, where *σ* = 0.1 (strong selection) and $D=\frac{{\displaystyle {\sum}_{r\in ER}}{\displaystyle {\sum}_{i=1}^L}\left|{a}_{r,i}-{a}_{r,i}^f\right|}{\left|ER\right|\cdot L}$ (*ER*: set of expressed receptors, |*ER*|: the number expressed receptors, *a*_*r*,*i*_: the *i* ^*th*^ entry of the amino acid sequence of receptor *r*, *a*_*r*,*i*_^*f*^: the *i* ^*th*^ entry of the amino acid sequence of the founder receptor *r*), which is the mean L1 distance from the founder amino acid sequence for all expressed receptors.

In preparation for the infection phase, two founder viruses are generated based on protein sequences from host individuals in order to guarantee a high initial transmission rate. Specifically, each founder virus is copied from a receptor protein sequence of a random host, then mutated using the virus protein mutation rate (*μ*_*vp*_ = 0.1 per virus protein). Although we tested a case of larger initial virus population size including a greater diversity of founder viruses, we could not find a significant difference from the small initial founder virus population case in terms of the infection strategy of the virus. Hence, in this study, we used two founder viruses for all simulations. Once the host-virus coevolution phase begins, the hosts are divided into susceptible and infected populations and the host population is no longer under stabilizing selection, as hosts need to acquire phenotypic variation to defend against virus entry. Initially all hosts are susceptible and as the founder viruses infect the healthy hosts, those hosts are moved to the infected population. Each individual in the infected group possesses the virus that caused the infection. From this point the population evolves under conditions of co-evolutionary selection and the size of the susceptible (S) and infected (I) groups is allowed to vary. The susceptible and infected population dynamics are inspired by the standard SIS model with births and deaths as shown in the following difference equations:

1 $$ \varDelta S=S\left(t+1\right)-S(t)=\eta \cdot b\cdot N(t)\cdot \left(1-\frac{N(t)}{K}\right)-\xi \cdot \frac{r}{N(t)}\cdot S(t)\cdot I(t)-{\lambda}_N\cdot S(t)+\gamma \cdot I(t) $$

2 $$ \varDelta I=I\left(t+1\right)-I(t)=\xi \cdot \frac{r}{N(t)}\cdot S(t)\cdot I(t)-\left({\lambda}_N+{\lambda}_D+\gamma \right)\cdot I(t) $$

where *N*(*t*) = *S*(*t*) + *I*(*t*),*b* = growth rate, *K* = carrying capacity, $\eta =\frac{\#\  of\  survived\  of fspring}{\#\  of\  of fspring\  candidates}$, *r* = contact rate, $\xi =\frac{\#\  of\  infections}{\#\  of\  contacts}$ (determined empirically, as described below), *r* ⋅ *ξ* = transmission rate, *λ*_*N*_ = natural death rate, *λ*_*D*_ = disease related death rate, *γ* = recovery rate. The main difference from the standard ODE SIS model is that *ξ* and *η* are determined by the individuals in the population and these parameter values can change as the population evolves. In our model, *ξ* and *η* are determined through a complex process that includes random sampling within the population and the evaluation of individual phenotypes. The transmission rate is frequency dependent (i.e., divided by *N*(*t*)), which assumes that a population occupies an area proportional to its size, i.e., per capita contact rate does not depend on population density, i.e. assuming a wide and unrestricted region affected by infectious viruses [42]. We also use standard assumptions of logistic population growth and that every offspring is initially susceptible. The difference equations dictate the number of offspring that need to be generated, the number of contact events between infected and susceptible hosts, host deaths, and recovered hosts at every time step, but because our model is individual-based, these numeric changes are applied to the actual populations as follows:

The growth term, $\eta \cdot b\cdot N(t)\cdot \left(1-\frac{N(t)}{K}\right)$, describes the number of offspring, which are generated via sexual or asexual reproduction and mutations in GRN and amino acid sequences are generated as described above. The term $b\cdot N(t)\cdot \left(1-\frac{N(t)}{K}\right)$ is the total number of offspring candidates who have the stable gene expression and express at least *N*_*ER*_ receptors. As candidates who have infected parents are less likely to survive, only a fraction of the candidates (*η*) can actually be added to the susceptible population. If phenotypes of the offspring candidates satisfy the criteria of expressing the minimal number (*N*_*ER*_) of receptor genes, and depending on the survival probability, the candidate may be added to the susceptible population. The survival probability is 1 if both parents are susceptible, *k*_*I*_ < 1 if both parents are infected, or $\frac{k_I+1}{2}$ if only one parent is infected. Therefore among the $b\cdot N(t)\cdot \left(1-\frac{N(t)}{K}\right)$ candidate offspring, only a fraction *η* of candidates can be added to the susceptible population when *k*_*I*_ is less than 1. Thus, the parameter *k*_*I*_ determines selection due to viral pathogenicity. For the infection term, the number of contacts is $\frac{r}{N(t)}\cdot S(t)\cdot I(t)$. Here, for each contact we choose a random pair of susceptible and infected individuals. Each infected host individual contains a single virus that caused the infection. With each host-virus contact event, the virus mutates the original amino acid sequence at the point of the infection with mutation rate, *μ*_*vp*_ = 0.1 per protein. The virus can bind a host receptor if the percentage of one-to-one amino acid pairs that match between the virus and the host receptor exceeds a matching threshold, *ϵ*_*seqM*_. If the virus can bind at least one of the expressed receptors on a susceptible host, then the infection proceeds and the individual moves from the susceptible to the infected population together with the virus that infected it, otherwise the susceptible individual remains in the susceptible population. Successive infection attempts by the same infected individual will involve new mutations with each host-virus contact occurs. Thus, virus transmission will depend on the coevolving host resistance and pathogen virulence. Also, note that the fraction of successful infections *ξ* in the Eqs. 1 and 2 is determined empirically, rather than as a given parameter.

### Parameters

There are parameters at both the level of population dynamics and at the individual level, i.e. governing the regulatory network and the protein sequences (Table 1). As described in the main text and in the figures, we tested a range of parameters including protein binding site amino acid sequence length (*L*), the minimum number of required expressed receptors (*N*_*ER*_), host protein mutation rate (*μ*_*hp*_), amino acid matching threshold for receptor binding ( *ϵ*_*seqM*_), offspring survival probability from both infected parents (*k*_*I*_) and disease-related death rate (*λ*_*D*_) to investigate the effect of parameter changes on host resistance evolution. Unless otherwise stated, in the main text figures we used the following parameters: for the population dynamics model, the number of simulations = 100, initial host population size *M*_*init*_ = 150, initial virus population size = 2, offspring survival probability from both infected parents *k*_*I*_ = 0.8, amino acid matching threshold for receptor binding *ϵ*_*seqM*_ = 90 %, carrying capacity *K* = 1000, growth rate *b* = 0.15, natural death rate *λ*_*N*_ = 0.09, disease-related death rate *λ*_*D*_ = 0.06, recovery rate *γ* = 0.2, host-virus contact rate *r* = 2. These parameters are chosen to make steady state host population size large enough to investigate evolutionary mechanisms. For the GRN and protein evolution model, virus protein mutation rate *μ*_*vp*_ = 0.1, the number of TFs *N*_*TF*_ = 5, network density *c* = 0.4, mutation rate per *W μ* = 0.1 with *ρ* = 0.028 and *ϕ* = 0.042 (*ϕ* + *δ* = 1). Note that *ϕ* + *δ* = 1, since for an interaction (*w*_*ij*_), deletion and modification are conditional on the interaction being nonzero value (*w*_*ij*_ ≠ 0). These individual level parameters are chosen based on our previous study [10].

**Table 1:** The list of model parameters

Parameter symbol	Description	Values
*L*	Protein binding site amino acid sequence length	5, 10, 15, 20, 25, 30
*μ*_*hp*_	Host protein mutation rate per a set of receptors	0.002, 0.01, 0.05
*μ*_*vp*_	Virus protein mutation rate	0.1
*N*_*TF*_	The number of transcription factor genes	5
*N*_*R*_	The number of receptor genes	5
*N*_*ER*_	The minimum number of required expressed receptors	1, 3
*ϵ*_*seqM*_	Amino acid matching threshold for receptor binding	90 %, 75 %
*k*_*I*_	Offspring survival probability from both infected parents	0.5, 0.8
*ξ*	$\frac{\#\ of\ infections}{\#\ of\ contacts}$	Self-determined during simulations
*η*	$\frac{\#\ of\ survived\ of fspring}{\#\ of\ of fspring\ candidates}$	Self-determined during simulations
*K*	Carrying capacity	1000
*M*_*init*_	Initial host population size	150
*b*	Growth rate	0.15
*λ*_*N*_	Natural death rate	0.09
*λ*_*D*_	Disease-related death rate	0.06
*γ*	Recovery rate	0.2
*r*	Host-virus contact rate	2
*c*	Network density	0.4
*μ*	Mutation rate per gene regulatory network	0.1
*ρ*	Conditional rate of interaction addition in gene regulatory network	0.028
*ϕ*	Conditional rate of interaction deletion in gene regulatory network	0. 042
*δ*	Conditional rate of interaction modification in gene regulatory network	0. 958
*σ*	Selection pressure	0.1
*a*	Gene expression mapping sigmoid function parameter	100

The list of model parameters at both the level of population dynamics and at the individual level in symbols with descriptions and parameter values used in this study

### Measure of unevenness among targeted receptors

Every 50 time steps after the coevolution phase has begun, we use the Gini coefficient to calculate unevenness in the targeted receptors among the newly infected hosts. Let *y*_*i*_(*i* = 1, …, *N*_*R*_) be the mean number of newly infected hosts who match their sequences to the *i* ^*th*^ receptor throughout the simulation. If these values are sorted in ascending order such that *y*_1_^′^ ≤ *y*_2_^′^ ≤ … ≤ *y*_*n* − 1_^′^ ≤ *y*_*n*_^′^, then the $\mathrm{Gini}\ \mathrm{coefficient} = \left(n+1-2\frac{{\displaystyle {\sum}_{i=1}^n}{y}_i^{\prime}\left(n+1-i\right)}{{\displaystyle {\sum}_{i=1}^n}{y}_i^{\prime }}\right)/n$. Gini coefficient is 1 for the maximum unevenness (inequality) and 0 for perfect evenness (equality).

### Measure of ability to switch multiple receptors using gene regulatory network rewiring

Every regulatory interaction in the GRN is mutated 50 times and we measure how often it switches expression of more than one gene. We then measure the average fraction of such perturbations that caused a multi-receptor expression switch over all regulatory interactions in the network for all susceptible individuals.

## Results

### Population dynamics of infection

**Fig. 1:**
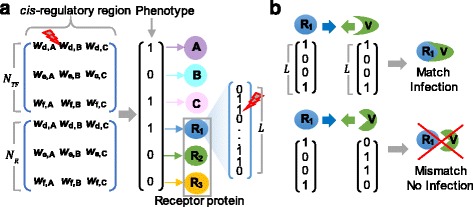
Diagram of gene regulatory network (GRN) and host-virus interaction scheme. **a** the GRN is composed of a transcription factor regulation sub-network and a receptor protein coding regulation sub-network. Mutations at the network level can be used to shut down the targetable receptor. Mutations at the protein level can result in a protein mismatch to block virus protein binding. **b** If more than *ϵ*_*seqM*_ % of amino acids are one-to-one matched, we assume the virus protein can bind to the matched receptor (top). If less than the threshold (*ϵ*_*seqM*_) are matched, we assume the virus protein fails to bind the receptor

For many infectious diseases, hosts never achieve long-term immunity due to rapid pathogen divergence. In particular, RNA viruses such as rhinoviruses and coronaviruses mutate so rapidly that even hosts that have recently recovered from an infection can become susceptible again to different strains of the same viruses circulating in the population. The Susceptible-Infectious-Susceptible (SIS) model is a simple infectious disease model that has been widely used to describe population dynamics for rapidly evolving pathogens and their target host populations [43, 44]. We introduce a model of host-virus coevolution that extends the gene regulatory network evolution model of gene regulatory network evolution, integrating it with a discretized form of the SIS model at the population level (see Methods). In our combined model, population sizes can vary, in contrast to the original gene regulatory network evolution model that considered a fixed population size. Since we preserve an explicit representation of each individual genotype in the population, we can observe the evolution of defense and infection mechanisms in both the host and pathogen populations. In its standard form, the SIS model uses fixed values to describe parameters such as the infection transmission rate. However, on evolutionary timescales, parameters such as host susceptibility and pathogen virulence are likely to vary over time and consequently key model parameters such as the transmissibility, *ξ*, will also change. In our model, each host genotype is represented explicitly with a gene regulatory network and the corresponding receptor protein sequences (Fig. 1). Each virus is represented explicitly with a receptor binding protein sequence, that will be compared to the host receptor sequences during contact (attempted infection) events (Fig. 1). Hence, rather than determining the rate of infection based on a fixed parameter, as in the standard SIS model, we allow the contacting host and pathogen phenotypes to determine infection events. Specifically, the key transmission parameter $\left(\xi =\frac{\#\  of\  infections}{\#\  of\  contacts}\right)$ that determines the infection rate (*r* ⋅ *ξ*) changes as both hosts and viruses evolve. Analytically, the steady state susceptible and infectious population sizes are $\tilde{S}=\frac{\delta_I}{r\cdot \xi}\cdot K\cdot \left\{1-\frac{1}{b\cdot \eta}\cdot \left({\lambda}_N+{\lambda}_D\left(1-\frac{\delta_I}{r\cdot \xi}\right)\right)\right\}$ and $\tilde{I}=\left(1-\frac{\delta_I}{r\cdot \xi}\right) \cdot K\cdot \left\{1-\frac{1}{b\cdot \eta}\cdot \left({\lambda}_N+{\lambda}_D\left(1-\frac{\delta_I}{r\cdot \xi}\right)\right)\right\}$ respectively when *r* ⋅ *ξ* ≠ 0 and $\frac{b\cdot \eta -{\lambda}_N}{\lambda_D}>1-\frac{\delta_I}{r\cdot \xi }>0$ where *δ*_*I*_ = *λ*_*N*_ + *λ*_*D*_ + *γ*. Different steady state values of *ξ* lead to different $\tilde{S}$ and *Ĩ* since these population sizes ultimately depend on the value of *ξ*. Since our main interest is the evolution of host resistance mechanisms, we only analyzed cases where the mean population size over time is greater than the initial susceptible population size (*M*_*init*_ = 150). In cases where the mean total population size < *M*_*init*_ (Additional file 2: Figure S1), we found that the susceptible population was too small to investigate and these cases mostly occur when the extremely infectious viruses appear which can spread widely and makes the host population sick.

**Fig. 2:**
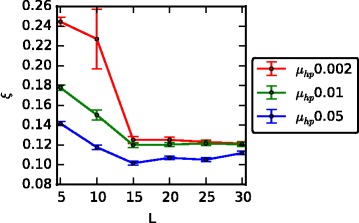
Transmissibility changes for different receptor binding complexity and host protein mutation rate. The mean transmissibility (*ξ*) for the last 250 time points (Error bar: one std. dev. over 100 simulations). *ξ* increases as the receptor binding complexity decreases (shorter *L*) in which case viruses can target multiple receptors and as the host protein mutation rate (*μ*_*hp*_) decreases which is due to the more limited speed of protein mutations to counteract the rapidly evolving viruses

We measured the steady state transmissibility (*ξ*), defined here as the mean value of *ξ* across the last 250 time points in each simulation, and considered how this measure changed under different conditions such as the protein binding sequence complexity (length, *L*), host protein mutation rate (*μ*_*hp*_), the number of required expressed receptors (*N*_*ER*_), the threshold above which the virus and receptor proteins are considered to have matched (*ϵ*_*seqM*_), the survival rate from infected parents (*k*_*I*_) and the disease-related death rate (*λ*_*D*_). As shown in Fig. 2, higher receptor binding sequence complexity (*L*) and higher host protein mutation rates (*μ*_*hp*_) tend to generate lower transmissibility *ξ* and are therefore disadvantageous to virus transmission. Similarly, when more receptors have to be expressed on the host cell surface (higher *N*_*ER*_), there are more ways in which viruses can attempt receptor binding and consequently, *ξ* tends to increase together with the number of required expressed receptor (*N*_*ER*_), at least when the receptor binding complexity is low (Additional file 3: Figure S2 a). For similar reasons, the transmissibility *ξ* also increases for lower matching threshold (*ϵ*_*seqM*_) value, such that when protein binding sequence complexity (*L*) is low, reducing the matching threshold (*ϵ*_*seqM*_) dramatically increases virus transmission whereas for complex receptor binding, it does not have an advantageous effect on *ξ* (Additional file 3: Figure S2 b). That transmissibility *ξ* increases only in the case of low complexity binding can be explained by the way viruses target host receptors, as explained in the next section. Intuitively, when a survival rate from infected parents (*k*_*I*_) is low, non-resistant offspring have much lower fitness (if infected) than resistant offspring, and thus resistant individuals should increase in frequency. This would actually tend to decrease *ξ* which is the opposite of what we observe. However, we found that in practice, it is more common for a low *k*_*I*_ value to cause population decay and a large decrease in the number of contacts between host and virus individuals as shown in (Additional file 4: Figure S3). A reduced number of contacts causes a larger decrease in the denominator of $\xi\ \left(\frac{\#\  of\  infections}{\#\  of\  contacts}\right)$, and therefore leads to a net increase in *ξ* (Additional file 3: Figure S2 c). The observation of higher *ξ* as a consequence of a high disease related death rate (*λ*_*D*_) is due to the same reason as for low *k*_*I*_ (Additional file 3: Figure S2 d). In sum, the virus transmissibility is dependent on various conditions for different underlying reasons. We now consider in greater detail why and how these variables affect the host and virus population dynamics and virus transmission.

### Host resistance strategy depends on the number of targeted receptors

Since receptor-virus protein binding enables virus entry and determines whether the infection succeeds, the virus’s ability to target multiple receptors and host’s ability to escape virus protein binding will have a significant impact on host resistance and viral pathogenicity. Hence we measured the number of targeted receptors across a variety of different conditions. We next show how the number of targeted receptors can change depending on the receptor binding complexity (protein sequence length, *L*), the number of required expressed receptors (*N*_*ER*_), protein binding threshold (*ϵ*_*seqM*_), the survival rate from infected parents (*k*_*I*_) and the disease-related death rate (*λ*_*D*_). As each simulation proceeded, we measured the frequency with which multiple receptors are targeted simultaneously and also used the Gini coefficient to measure the unevenness in the distribution of targeted receptors among the newly infected hosts throughout the simulation (see Methods). Thus, for example, when the frequency of multi-receptor matching is low, this indicates that mostly a single receptor is being targeted by the virus. However, this does not guarantee that the virus population targets the same specific receptor or whether different subpopulations are targeting distinct receptors. In this case, when the Gini coefficient of targeted receptors is high, this indicates that all viruses target a common receptor and when the Gini coefficient is low, this implies that the matched receptor for each host is different and that viruses have diversified into subpopulations by targeting different receptors.

**Fig. 3:**
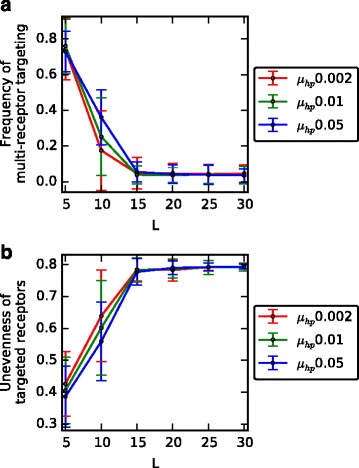
Two different virus infection strategies: Targeting a specific receptor or non-specific multiple receptors. **a** The fraction of time points that multiple receptors are targeted simultaneously and **b** the Gini coefficient of the frequency of targeted receptors for different receptor binding complexities (*Ls*) (Error bar: std. dev. over 100 simulations). A lower Gini coefficient (close to zero) indicates evenness and one that is close to one indicates inequality. As the receptor binding complexity increases (longer *L*) viruses target a specific receptor and do not change the target receptor over time

When binding complexity (*L*) is low, viruses can target different receptors by means of a few amino acid mutations, whereas when receptor binding complexity is high, targeting multiple receptors is more difficult since the different receptors are likely separated by more mutations. Hence, as shown in (Fig. 3), when *L* is short, multiple receptors are often targeted simultaneously and the frequency of each receptor being targeted is not highly variable (low Gini coefficient). Considering this, more permissive receptor binding (lower *ϵ*_*seqM*_), increases the chances for multiple receptor targeting when *L* is short (Additional file 5: Figure S4 c, d). On the other hand, when binding complexity is high, a single receptor is usually targeted and the Gini coefficient is close to 1 indicating there are usually one or two dominant targeted receptors (Fig. 3). Furthermore, in this case, reducing the receptor binding threshold does not help viruses target multiple receptors (Additional file 5: Figure S4 c, d). These results indicate that for complex receptor binding, one or two receptors are targeted for virus entry and that there is no switch from one targeted receptor to another (Fig. 3). Based on this observation, as expression of more distinct receptors is required (higher *N*_*ER*_), multiple receptors can be targeted and at the same time the Gini coefficient decreases only when receptor binding complexity is low (short *L*). On the other hand, when receptor binding is complex (long *L*), increasing *N*_*ER*_ does not allow more receptors to be targeted by viruses (Additional file 5: Figure S4 a, b). Hence the number of required expressed receptors only impacts the strategy of the virus when the receptor binding is less complex (short *L*). Interestingly, the survival rate of offspring from infected parents also affects how the viruses target receptors. As we explained in the previous section, a low survival rate from infected parents (*k*_*I*_) causes the host population to become sick (the mean host population size is less than the initial population and the population is composed of more infected hosts than healthy hosts) and thus the population size decays. Consequently, as shown in Fig. 6d, e and f, we observe that variation within the host population decreases, suggesting that viruses will need to specialize on binding to specific receptors (Additional file 5: Figure S4 e, f). Specific receptor targeting as a consequence of high disease related death rate (*λ*_*D*_) arises for the same reason as for low *k*_*I*_ (Additional file 5: Figure S4 g, h). We tested the effect of diversity in the initial virus population on the number of targeted host proteins. We compared a case with a highly diverse initial virus population to the default case of two initial viruses. Thus, given an initial population of 15 distinct founder viruses, each three viruses were chosen to bind a distinct host receptor. With *L* = 30, *μ*_*hp*_ = 0.002 and *N*_*R*_ = 5, all virus strains except one went extinct. In this case, the frequency of multi-receptor targeting was 0.04 ± 0.04 and unevenness of targeting receptors (Gini coefficient) was 0.793 ± 0.009 which is close to the values for the 2 founder virus case. Even with *L* = 10, *μ*_*hp*_ = 0.002 and *N*_*R*_ = 5, we could not find a significant difference from the 2 founder case. Here, the frequency of multi-receptor targeting was 0.16 ± 0.14 and unevenness of targeting receptors (Gini coefficient) was 0.70 ± 0.08. In sum, receptor binding complexity (*L*) affects viruses by determining the variety of targetable receptors, although this also is dependent on parameters such as *N*_*ER*_ and *ϵ*_*seqM*_. Also indirect causality between host population diversity and parameters, *k*_*I*_ and *λ*_*D*_ has an influence on the specificity of targetable receptors. So far, we considered how viruses behave and choose infection strategies for different conditions. We next explore how hosts react to virus infection strategies differently depending on the various environments.

### Evolved preference for resistance using network rewiring

**Fig. 4:**
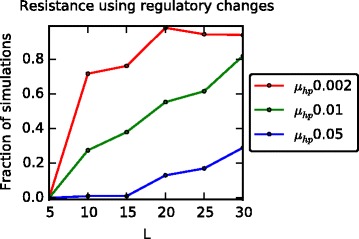
Preference for resistance using gene regulatory network (GRN) rewiring rather than protein mutations. The fraction of simulations where GRN rewiring strategy is used more often than protein binding site change for successful resistance under different protein binding complexities (*Ls*) and host receptor sequence mutation rates (*μ*_*hp*_). In a more complex receptor binding system, hosts tend to select the GRN rewiring strategy more often than the protein mutation strategy due to the single receptor targeting infection strategy. Since low *μ*_*hp*_ means a lower rate of protein mutations to counteract the rapidly evolving viruses, hosts tend to favor a protein mutation strategy less

Hosts can adopt two different resistance strategies in the model: 1) Gene regulatory network rewiring to switch a targeted receptor off and 2) protein binding site changes to block protein binding to a targetable receptor. Here we consider how hosts balance the usage of these two strategies and what conditions determine their relative preference. At each time step the most frequently targeted receptor is identified among the set of newly infected hosts and from here we measure how often successful resistance events use network rewiring to shut down the most targetable receptor rather than protein sequence changes. We proceed by counting the fraction of hosts who resisted successfully and that do not express the most frequently targeted receptor. If there are multiple equally frequent most targeted receptors, we use the mean frequency across those receptors. The fraction of resisted hosts using network rewiring was measured at every time point. We then accumulated these measurements over all time points throughout the simulation and if the overall use of network rewiring resistance was higher than protein level resistance, we counted the simulation as preferential to rewiring. We subsequently measured the fraction of simulations for which this occurred to quantify the relative use of rewiring across many simulations. Using this measure, we find that GRN rewiring is preferentially used as protein binding complexity increases (Fig. 4). This outcome relates to the number of targeted receptors since when protein binding is more complex, the virus most often targets a single receptor and therefore down-regulating the targetable receptor is usually an effective strategy. Conversely when protein binding is low complexity, viruses are able to enter the host cell by binding multiple receptors and therefore rewiring is a less effective host strategy for resistance. As the host protein mutation rate (*μ*_*hp*_) decreases, hosts also use GRN rewiring more often due to the reduced ability to catch up with the relatively fast-evolving virus proteins (Fig. 4). As we increase the number of receptors that need to be expressed (*N*_*ER*_) then combinatorially there are fewer possible phenotypes for a given number of required receptors, and viruses have more chances to bind to the different receptors so that the frequency of resistance using GRN rewiring decreases (Additional file 6: Figure S5 a). Reducing the protein matching threshold also favors the protein interaction level (Additional file 6: Figure S5 b). Lastly, at low survival rate (*k*_*I*_) from infected parents and at high disease related death rate (*λ*_*D*_), viruses tend to target more specific receptors, which is due to population size decay and low population diversity (Additional file 5: Figure S4 e ~ h). In fact, as shown in (Additional file 7: Figure S6 g, i), the *potential* for resistance (which will be explained in the following paragraph) via network rewiring increases. However, the small population size and low variation do not allow this potential to be realized. This explains the apparently contradictory result of (Additional file 6: Figure S5 c, d), where the observed (as opposed to potential) number of resistance events occurring via GRN decreases when *k*_*I*_ is low but also when *λ*_*D*_ is high. Hence, unlike with *L*, *N*_*ER*_ and *ϵ*_*seqM*_, we observed that low *k*_*I*_ and high *λ*_*D*_ did not promote resistance via network rewiring (Additional file 6: Figure S5 c, d). In sum, hosts choose a resistance mechanism depending on the virus infection strategy and their defense ability relative to viruses (how fast they react to the fast evolving viruses). In the next section, we consider the temporal dynamics of hosts with respect to regulatory network and receptor protein binding evolution.

### Evolutionarily gained potential to switch from infectious to resistance using GRN rewiring and protein mutations

In the previous section, we showed that hosts determine the resistance strategy between GRN rewiring and protein binding site mutation depending on factors such as binding site complexity and mutation rate relative to that of the virus. We now consider the evolution of the potential within the population to resist future virus contact events. For each virus in the infected group, we selected all susceptible hosts in the population that can be potentially infected by that virus and measure how efficiently each host can avoid infection via a random mutation either in its GRN or in protein binding sites. Every regulatory interaction in the GRN was mutated multiple times and we then measured how often it switched to becoming resistant as a consequence of these network perturbations. Similarly, for each matched receptor, we mutate the receptor using the host protein mutation rate at each site (as would occur during the simulation) and measured the average fraction of such perturbations that caused a switch to resistance. The reason for using the same protein mutation rate that is used within the simulation rather than a single random amino acid mutation for the perturbation is that the impact of a single site amino acid mutation differs depending on the protein binding site length (*L*). For example, when *L* is long, a chance of switching from infectious to resistible is very low, whereas when *L* is short, a host can easily switch from infectious to resistible.

**Fig. 5:**
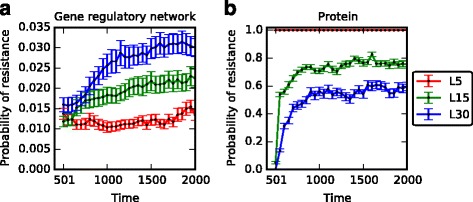
Trade-offs in the resistance potential between the gene regulatory network and receptor proteins. For the susceptible host population, the ability to resist using **a** GRN rewiring and **b** protein binding site changes is measured for different receptor binding complexities (Error bar: std. dev. over 100 simulations). As the receptor binding complexity increases, hosts increase evolutionary potential more on the GRN while decreasing it on receptor proteins (*μ*_*hp*_ = 0.01, *N*_*ER*_/*N*_*R*_ = 3/5, *ϵ*_*seqM*_ = 90 %, *k*_*I*_ = 0.8)

For resistance acquired via regulatory rewiring, the ability to resist increases only when the protein complexity is high (Fig. 5 a blue and green lines), while it does not increase when the protein binding complexity is low (red line). It is plausible that when the protein binding complexity is low, since network rewiring is not a good resistance strategy (Fig. 4) due to multiple receptor binding site matches by viruses (Fig. 3), it is unnecessary for individuals to evolve network rewiring potential and for this reason few perturbations are expected to change receptor gene expression to switch the targetable receptor off. In contrast, when the protein binding complexity is high so that the targeted receptor is specialized to one receptor (Fig. 3) and switching targetable receptor off by network rewiring is adopted by hosts (Fig. 4), hosts evolve the potential to resist by network rewiring. In contrast, for resistance via protein mutations, we observed that under all conditions hosts rapidly evolve the ability to acquire resistance via protein binding site changes (Fig. 5b and Additional file 7: Figure S6) because the protein binding site mutations can directly affect virus protein binding.

We also observed that there is an apparent tradeoff in that, as the resistance ability via rewiring increases (Fig. 5a) with receptor binding complexity, the ability to resist using binding site mutations decreases (compare order of curves in Fig. 5a vs Fig. 5b). The complexity of the protein-protein interaction appears therefore to be an important factor driving the transition toward resistance using regulation and thus leading to higher GRN complexity. As expected, when the protein mutation rate is low, hosts will use GRN rewiring more for resistance as a consequence of the limited capacity for protein mutations to coevolve with the viruses (Additional file 7: Figure S6 a, b). The ability to resist using network rewiring also depends on the number of required expressed receptors (*N*_*ER*_). As more receptors are required to be expressed (*N*_*ER*_), viruses have a greater probability of targeting more than one receptor. Hence, as shown above in (Additional file 6: Figure S5 a), the fraction of simulations where GRN rewiring is used in preference to protein mutation decreases for higher values of *N*_*ER*_. However, for the same reason, hosts are under pressure to evolve the ability to resist using network rewiring more when more receptors are required to be expressed (Additional file 7: Figure S6 c, d). In the (Additional file 5: Figure S4 c, d), in higher matching threshold (*ϵ*_*seqM*_) condition, viruses are not able to target multiple receptors and the fraction of simulations where GRN rewiring is preferentially used also increases (Additional file 6: Figure S5 b). Consequently high *ϵ*_*seqM*_ results in evolution of the potential to resist infection using GRN (Additional file 7: Figure S6 e, f). A lower survival rate from infected parents induces viruses to target specific receptors (Additional file 5: Figure S4 e, f). Therefore, for such viruses, hosts are evolved to increase the ability to resist using GRN rewiring to shut down the targetable receptor (Additional file 7: Figure S6 g, h).

So far, we explored various conditions that can promote the evolution of the ability to resist using GRN rewiring. Interestingly, receptor binding complexity balances the usages of GRN rewiring vs amino acid mutations for resistance. Resistance via protein binding site mutation is much higher than that using network rewiring under all conditions. This may explain why receptor binding site mutations have been reported often for virus entry defense mechanisms in contrast to resistance via regulatory changes.

### Genetic diversity and host range

**Fig. 6:**
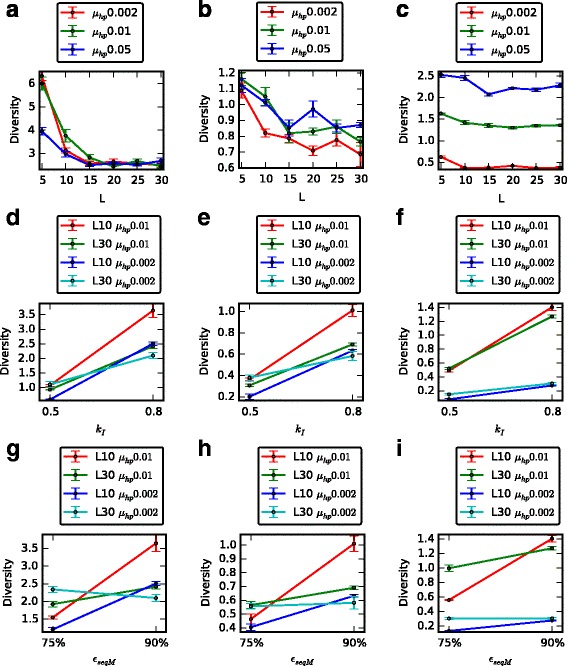
Host range measured by infected host population’s genetic diversity under different conditions. The first column is the gene regulatory network diversity, the second column is the phenotype diversity and the last column is the receptor protein sequence diversity. Viruses become specialists when receptor binding complexity (*L*) increases (**a**, **b**, **c**), survival rate for offspring from infected parents (*k*_*I*_) decreases (**d**, **e**, **f**) and amino acid matching threshold for protein binding (*ϵ*_*seqM*_) decreases (**g**, **h**, **i**). For low *ϵ*_*seqM*_ and *k*_*I*_, population dynamics generally follows that shown in Additional file 2: Figure S1 b. Hence, in d ~ i) we considered all 100 simulations for measuring the genetic diversity

In many previous studies it has been shown that antagonistic coevolution between host and pathogen populations correlates with increased genetic diversity [13, 45]. We checked that the diversity of the regulatory network, the phenotype and the protein sequence all increase throughout the coevolution phase (Additional file 8: Figure S7). To quantify diversity we used the Margalef index [46], an ecological measure of biodiversity that takes into account the expected increase in species sampled as a consequence of increased sample size $\left(\frac{\mathrm{the}\ \mathrm{number}\ \mathrm{of}\ \mathrm{genetic}\ \mathrm{variants}-1}{ \ln \left(\mathrm{total}\ \mathrm{number}\ \mathrm{of}\ \mathrm{individuals}\right)}\right)$. After we simplified each GRN using the sign of each interaction matrix entry (e.g., −0.8 to −1 and +0.8 to 1), we measured the GRN diversity of a susceptible host group as $\frac{\mathrm{the}\ \mathrm{number}\ \mathrm{of}\ \mathrm{distinct}\ \mathrm{GRNs}-1}{ \ln \left(\mathrm{susceptible}\ \mathrm{individuals}\right)}$. We found that diversity of GRNs, phenotypes and receptor protein sequences all increased throughout the coevolutionary phase, showing that coevolution between hosts and viruses is an important factor in producing genetic diversity. We also used the Margalef index to quantify the genetic diversity of the infected group to estimate virus host range. We compared the diversity over the last 250 time steps in intervals of 50-time steps to identify variables affecting host range and under what conditions pathogens evolve as specialists or generalists (Fig. 6). We observed that pathogens become either specialists or generalists dependent primarily on three parameters: protein binding complexity, survival rate for offspring from infected parents, and the matching threshold. For example, as receptor binding complexity increases, viruses tend to become specialists, which directly relates to the number of targeted receptors due to the difficulty in this case for binding multiple receptors (Fig. 6a ~ c). Also a lower survival rate for offspring from infected parents narrows the host range and leads viruses to become specialists because this condition causes the host population size to decay and thus reduces variations within the host population (Fig. 6d ~ f). For the same reason, since a low matching threshold is beneficial for virus entry when the binding complexity is low (short *L*), viruses become specialists (Fig. 6g ~ i).

## Discussion

We showed that regulatory changes can be used to suppress expression of cell surface receptor genes leading to a blocking of virus entry. Changes in the expression of virally-targeted receptors has been shown to block virus transmission experimentally, for example, in both dengue virus (DENV) [25] and Hepatitis C virus (HCV) [24], siRNAs can be used to eliminate cell surface receptors and suppress virus entry and infection. At the same time, specific receptors can be intentionally expressed in the context of tumor gene therapy, for example, allowing adenovirus vectors to be used [31, 32] to deliver apoptosis-activating genes to kill tumor cells.

Two mechanisms of resistance were addressed in our model: rewiring of gene regulatory networks and receptor binding site mutations. The balance in usage between these two mechanisms depends on various conditions. As the protein-protein interaction at the cell surface increases in complexity (in our model represented by the binding site length), viruses tend to target a specific receptor and hosts preferentially use network rewiring more often than receptor amino acid changes. In contrast, when the receptor binding site has lower complexity, viruses are able to enter via multiple receptors and hosts evolve receptor amino acid changes to escape viral protein binding. One can ask why is it that in nature, examples of resistance via receptor amino acid mutations appear to be more common than network rewiring? In the examples of dengue virus (DENV) and hepatitis C virus (HCV) resistance through experimentally-induced receptor down-regulation it was shown that, since there several alternative receptors expressed on the cell surface that viruses can use to enter host cells, multiple inhibitory siRNAs for different receptors worked better than a single siRNA for one receptor, although both studies showed that it was difficult to block infection completely [24]. Thus, for example, HCV can enter human liver cells via several cell surface receptors including CD81 tetraspanin, claudin1(CLDN1), low density lipoprotein receptor receptor (LDLR) and scavenger receptor class B type 1 (SR-B1). In our model, when receptor binding has low complexity, multiple receptors are targeted by viruses and receptor amino acid mutations are used preferentially over network rewiring. Given this observation, the capability of viruses to use alternative receptors for host cell entry is a plausible explanation of why resistance using network rewiring changes is difficult in practice. Another possible reason for more frequent protein level resistance could be related to the level of functional redundancy among receptors. Higher *N*_*ER*_ indicates less functional redundancy among receptors, and we found that protein level resistance was favored for higher *N*_*ER*_ (Additional file 6: Figure S5a). Although functional redundancy is often observed in receptors such as nectin and chemokine receptors as described in Introduction, it is plausible that viruses evolve to target receptors whose absence cannot be compensated for, so that hosts have to express all (or nearly all) required receptors for their normal function, which makes it difficult to use network level resistance.

In order to investigate the importance of including the complex GRN for controlling receptor gene expression, we compared our model with one that did not contain gene regulatory interactions for receptor coding genes. We designed this model by using a diagonal matrix regulatory network both for TF genes and for the receptor coding genes. Complex gene regulation by TFs were removed by having a diagonal matrix with 1 s for the regulatory gene network. To satisfy the minimum number of required expressed receptors (*N*_*ER*_/*N*_*R*_ = 3/5), we set the initial density of non-zeros on the diagonal for the receptor coding genes with probability 0.7. Here, mutations can occur only on the diagonal of receptor coding genes and no regulation from other genes is possible. Compared to this model, the benefit of having a complex GRN is that the network is capable of evolving increased potential for resistance using network rewiring as shown in Fig. 5a for complex protein binding (long *L*), as an example. Here, in the case of complex protein binding where a specific receptor is targeted, it is not possible for the potential for resistance to change because there is only a single entry on the diagonal which can change the expression of the targeted receptor. We compared the preference for GRN level resistance between these two models. We found that the preference of GRN rewiring decreased for the model without gene regulatory interactions (Additional file 9: Figure S8a). Furthermore, in order to express at least *N*_*ER*_ receptors for the normal host cell function, down-regulating a receptor gene for resistance can be deleterious, and therefore, hosts need to be able to change the expression of multiple receptors simultaneously, in particular to compensate for receptor down-regulation. We found that the systems with complex GRNs evolve the ability to switch the expression of multiple receptors (Additional file 9: Figure S8b and Methods), whereas without the GRNs, multiple receptor expression change is impossible given a single mutation.

Although defending from infection at the level of virus entry would appear to be an effective resistance mechanism, the host evolution rate is usually too slow relative to most virus populations and furthermore, viruses are often capable of entering host cells via interaction with multiple receptors. For these reasons, host strategies may have evolved preferentially to allow viruses to enter cells but to focus defense mechanisms to the post-entry stage by evolving innate and adaptive immune systems. For example, a previous study of North American house finches showed rewiring of gene regulatory networks to up-regulate immune related genes in a relatively short timespan of just 12 years [7].

In addition to network rewiring and receptor amino acid mutations, mutations causing premature stop codons can be used by hosts to block virus entry. CCR5 (CC-chemokine receptor-5) is a co-receptor for HIV entry that facilitates virus entry. A CCR5 allele carrying a 32-bp deletion (ccr5Δ32) in the open reading frame generates a premature stop codon leading to an inactive receptor protein [47, 48]. Homozygous ccr5Δ32/ccr5Δ32 carriers show high immunity to HIV infection and heterozygous wt/ccr5Δ32 carriers show partial resistance to HIV cell entry or delayed progression of the disease. A similar example is an allele of the TVB^R^ receptor involving a 4-bp insertion which contains a stop codon resulting in protection against Avian Sarcoma and Leukosis Virus (ASLV) entry in chicken [49]. Of note is that even though these stop codon-containing alleles can block virus entry, they work effectively only in homozygous form, in contrast to alleles encoding regulatory repression, which may be effective in single copy form.

## Conclusions

Entry to the cell is the first step in all virus infections. Evolving barriers to infection at the level of entry to the host cell can become an effective resistance mechanism. Although many examples of defense mechanisms have been reported that are based on disruption to cell surface receptor binding sites due to copy number variation and mutations producing stop codons have been reported, examples of resistance by gene regulatory changes in receptor expression levels are less commonly observed. We built a host-virus coevolution model where hosts are represented using both receptor amino acid sequences and gene regulatory networks (GRNs) that control expression of the cell surface receptor genes. We explored a range of evolutionary conditions that might determine the balance of host resistance mechanisms at the GRN level compared to protein interaction level. We observed that the complexity, or length, of the receptor binding site (*L*) is one of the key factors that have a significant impact on both the infection strategy of the virus and resistance mechanism of the host. When *L* is short, viruses evolved to be generalists and target multiple receptors for cell entry. In this case hosts evolve to a counter-strategy that uses binding site mutations to defend against virus protein binding. In contrast, when *L* is long, viruses evolve to be specialists and focus on targeting one particular receptor, whereas hosts evolve a counter strategy at the network level that uses regulatory changes to turn off the expression of the targeted receptor. Considering examples of virus entry such as hepatitis C virus, where viruses can make use of multiple receptors for entry to the cell, it is plausible that viruses predominantly evolve low complexity receptor binding and that in these cases hosts evolve to use protein binding level resistance mechanisms rather than GRN level mechanisms.

## Additional files

Additional file 1: C++ simulation source codes. (ZIP 59 kb)
Additional file 2: Figure S1. Two different types of susceptible and infectious population dynamics. Typical population dynamics of a) healthy population case where the mean host population size is greater than the initial host population size and b) sick population case where the mean host population size is less than the initial population and the population is composed of more infected hosts than healthy hosts. (*L* = 10, *N*_*ER*_ = 3, *μ*_*hp*_ = 0.002, *ϵ*_*seqM*_ = 75 %, *k*_*I*_ = 0.8). (PDF 154 kb)
Additional file 3: Figure S2. Transmissibility changes for different conditions. The mean transmissibility (*ξ*) for the last 250 time points (Error bar: one std. dev. over 100 simulations). a) *ξ* increases as the number of required receptor expression (*N*_*ER*_) increases when the binding complexity (*L*) is low. For low receptor binding threshold (*ϵ*_*seqM*_), low survival rate from both infected parents (*k*_*I*_) and high disease related death rate (*λ*_*D*_), population dynamics generally follows that shown in Additional file 2: Figure S1 b. Hence, in b), c) and d) we considered all 100 simulations for the comparison of mean *ξ* values. *ξ* increases as (b) the receptor binding site matching threshold (*ϵ*_*seqM*_) decreases, as (c) the survival rate from both infected parents (*k*_*I*_) decreases and as (d) disease related death rate (*λ*_*D*_) increases. (PDF 215 kb)
Additional file 4: Figure S3. The number of contacts between host and parasite populations for different offspring survival rate from infected parents. The number of contacts between host and parasite populations decreases when offspring survival rate from infected parents (*k*_*I*_) is low (Error bar: one std. dev. over 100 simulations). (PDF 49 kb)
Additional file 5: Figure S4. Viruses change their receptor targeting strategy under different conditions. The first column is the fraction of time points that multiple receptors are targeted simultaneously and the second column is the Gini coefficient of the frequency of targeted receptors (Error bar: one std. dev. over 100 simulations). a, b) When the binding complexity is low, a greater required number of expressed receptors (*N*_*ER*_) causes viruses to target multiple receptors simultaneously. However, when the binding complexity is high, a higher required number of expressed receptors does not change the targeting to a multiple receptor binding strategy. For low receptor binding threshold (*ϵ*_*seqM*_) and survival rate from both infected parents (*k*_*I*_), population dynamics generally follows the trend shown in Additional file 2: Figure S1 b. Hence, in c ~ h) we considered all 100 simulations for the comparison of the fraction of time points that multiple receptors are targeted simultaneously and the Gini coefficient of the frequency of targeted receptors. c, d) The low amino acid matching threshold for the receptor binding (*ϵ*_*seqM*_) facilitates viruses to target multiple receptors. e, f) The low survival rate of an offspring from both infected parents results in viruses targeting more specific receptors for more robust receptor binding. g, h) The high disease related death rate (*λ*_*D*_) causes more specialized receptor targeting. (PDF 473 kb)
Additional file 6: Figure S5. Preference for resistance using gene regulatory network (GRN) rewiring to protein mutations under different conditions. The fraction of simulations where GRN rewiring strategy is used more often than the protein binding site change strategy for resistance for different a) required number of expressed receptors (*N*_*ER*_), b) amino acid matching threshold for the receptor binding (*ϵ*_*seqM*_), c) survival rate from both infected parents (*k*_*I*_) and d) disease related death rate (*λ*_*D*_). For low *ϵ*_*seqM*_, *k*_*I*_ and *λ*_*D*_, the population dynamics generally follows that shown in Additional file 2: Figure S1 b. Hence, in b, c, d) we considered all 100 simulations for the comparison of the preference for resistance using GRN rewiring to protein mutations. a) As more receptors are required to be expressed (higher *N*_*ER*_), hosts preferentially use GRN rewiring less often than protein mutations. b) When the binding complexity is low, for lower amino acid matching threshold for the receptor binding (*ϵ*_*seqM*_), hosts do not preferentially select GRN rewiring strategy. c) When *k*_*I*_ is low, hosts does not favor the GRN rewiring strategy. d) When the disease related death rate (*λ*_*D*_) is high, hosts hosts less favor the GRN rewiring strategy for resistance. (PDF 208 kb)
Additional file 7: Figure S6. Evolutionary potential for resistance in the gene regulatory network and receptor proteins for different conditions. For susceptible host population, the ability to resist using GRN rewiring (1st column) and protein binding site changes (2nd column) is measured for different a, b) host protein mutation rates (*μ*_*hp*_), c, d) number of required expressed receptors (*N*_*ER*_), e, f) amino acid matching threshold for the receptor binding (*ϵ*_*seqM*_), g, h) survival rate from both infected parents (*k*_*I*_) and i, j) disease related death rate (*λ*_*D*_) (Error bar: std. dev. over 100 simulations). For low *ϵ*_*seqM*_ and *k*_*I*_, population dynamics generally follows that of Additional file 2: Figure S1 b. Hence, in e ~ h) we considered all 100 simulations for the comparison of the resistance potentials. a, b) For lower *μ*_*hp*_, hosts evolve a GRN based strategy (*L* = 30, *μ*_*hp*_ = 0.01, *ϵ*_*seqM*_ = 90 %, *k*_*I*_ = 0.8). c, d) When expression of more receptors is required, hosts evolve the potential for resistance using GRN rewiring to higher level. (*L* = 30, *N*_*ER*_/*N*_*R*_ = 3/5, *ϵ*_*seqM*_ = 90 %, *k*_*I*_ = 0.8), e, f) When receptor binding is simple (short *L*), for reduced *ϵ*_*seqM*_ hosts does not necessarily evolve the potential for a GRN rewiring strategy (*L* = 10, *μ*_*hp*_ = 0.002, *N*_*ER*_/*N*_*R*_ = 3/5, *k*_*I*_ = 0.8). g, h) Selection pressure triggered by the low *k*_*I*_ evolves the potential for GRN rewiring strategy (*L* = 30, *μ*_*hp*_ = 0.002, *N*_*ER*_/*N*_*R*_ = 3/5, *ϵ*_*seqM*_ = 90 %). i, j) The potential for resistance using network rewiring increases both for low and high diseases related death rates (*λ*_*D*_). (PDF 2128 kb)
Additional file 8: Figure S7. Increased genetic diversity in the gene regulatory networks, phenotypes and receptor proteins. Genetic diversity is measured using the Margalef index (see the last section in Results). a) whole GRNs (blue), transcription factor regulation sub-networks (red), receptor regulation sub-networks (green) of susceptible hosts. b) Phenotypes (gene expression levels) of susceptible populations. c) Receptor sequence of susceptible populations. (PDF 461 kb)
Additional file 9: Figure S8. The effect of having a complex gene regulatory network (GRN) for controlling receptor gene expression. a) Preference for resistance using GRN rewiring to protein mutations decreases when there are no regulatory interactions between genes (without regulatory interactions in the gene network) (*N*_*ER*_/*N*_*R*_ = 3/5, *ϵ*_*seqM*_ = 90 %, *k*_*I*_ = 0.8). b) The ability to switch the expression of multiple receptors with a complex GRN. The probability of multiple receptor gene expression switching (see Methods) increases during host-virus coevolution (*L* = 30, *μ*_*hp*_ = 0.01 and 0.002, *ϵ*_*seqM*_ = 90 %, *k*_*I*_ = 0.8). (PDF 245 kb)

## Abbreviations

CAR: Coxsackie and Adenovirus Receptor
CCR5: CC-chemokine receptor-5
GRN: Gene regulatory network
HCV: Hepatitis C virus
I: The size of infected
MMTV: Machupo virus
PPI: Protein-protein interactions
S: The size of susceptible
SIS: Susceptible-Infected-Susceptible
TF: Transcription factor
TfR1: Transferrin Receptor-1
