# Supplementary figures and images for: Potential for evolution of complex defense strategies in a multi-scale model of virus-host coevolution
Source: BMC Evol Biol. 2016 Oct 26;16:233. doi: 10.1186/s12862-016-0804-z (PMC5080737; doi:10.1186/s12862-016-0804-z)

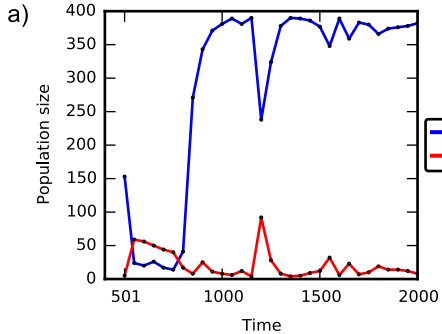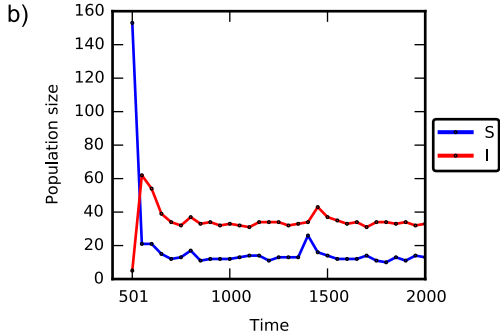

Supplement: Additional file 2: Figure S1. — Two different types of susceptible and infectious population dynamics. Typical population dynamics of a) healthy population case where the mean host population size is greater than the initial host population size and b) sick population case where the mean host population size is less than the initial population and the population is composed of more infected hosts than healthy hosts. (L = 10, N ER = 3, μ hp = 0.002, ϵ seqM = 75 %, k I = 0.8). (PDF 154 kb) [file 12862_2016_804_MOESM2_ESM.pdf]

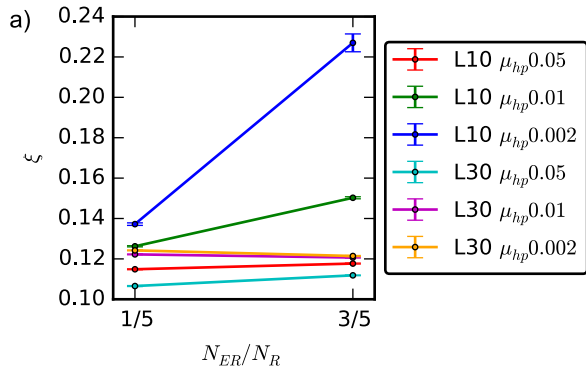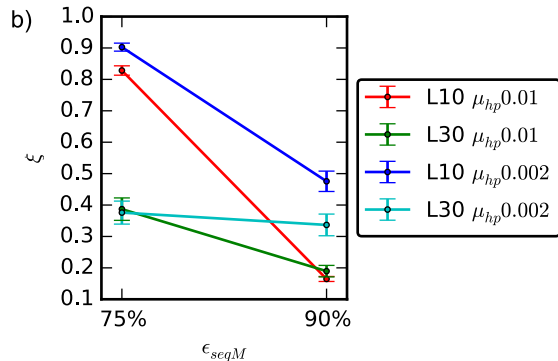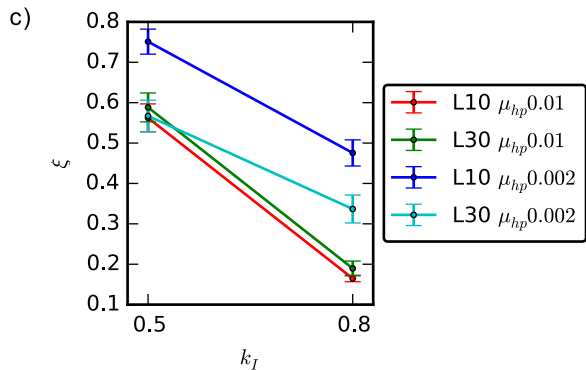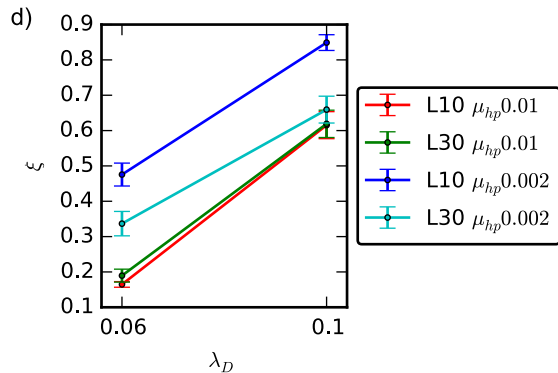

Supplement: Additional file 3: Figure S2. — Transmissibility changes for different conditions. The mean transmissibility (ξ) for the last 250 time points (Error bar: one std. dev. over 100 simulations). a) ξ increases as the number of required receptor expression (N ER) increases when the binding complexity (L) is low. For low receptor binding threshold (ϵ seqM), low survival rate from both infected parents (k I) and high disease related death rate (λ D), population dynamics generally follows that shown in Additional file 2: Figure S1 b. Hence, in b), c) and d) we considered all 100 simulations for the comparison of mean ξ values. ξ increases as (b) the receptor binding site matching threshold (ϵ seqM) decreases, as (c) the survival rate from both infected parents (k I) decreases and as (d) disease related death rate (λ D) increases. (PDF 215 kb) [file 12862_2016_804_MOESM3_ESM.pdf]

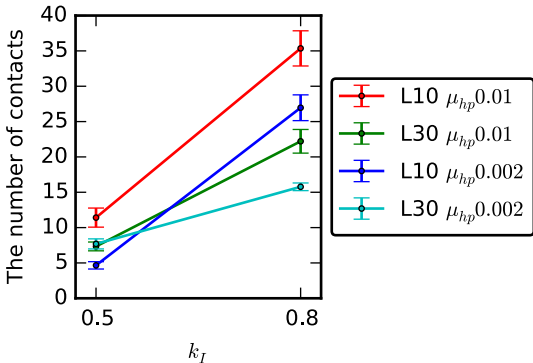

Supplement: Additional file 4: Figure S3. — The number of contacts between host and parasite populations for different offspring survival rate from infected parents. The number of contacts between host and parasite populations decreases when offspring survival rate from infected parents (k I) is low (Error bar: one std. dev. over 100 simulations). (PDF 49 kb) [file 12862_2016_804_MOESM4_ESM.pdf]

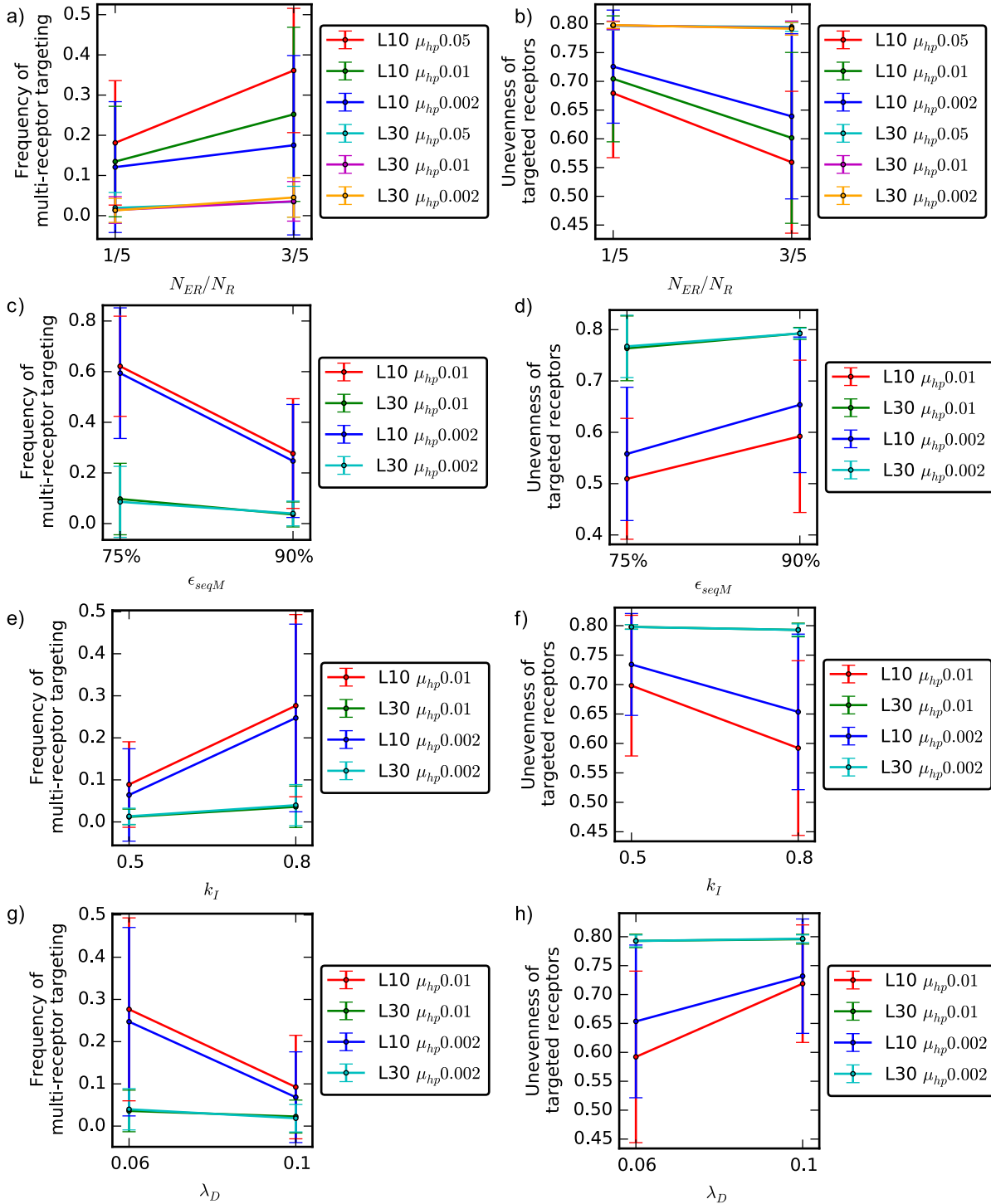

Supplement: Additional file 5: Figure S4. — Viruses change their receptor targeting strategy under different conditions. The first column is the fraction of time points that multiple receptors are targeted simultaneously and the second column is the Gini coefficient of the frequency of targeted receptors (Error bar: one std. dev. over 100 simulations). a, b) When the binding complexity is low, a greater required number of expressed receptors (N ER) causes viruses to target multiple receptors simultaneously. However, when the binding complexity is high, a higher required number of expressed receptors does not change the targeting to a multiple receptor binding strategy. For low receptor binding threshold (ϵ seqM) and survival rate from both infected parents (k I), population dynamics generally follows the trend shown in Additional file 2: Figure S1 b. Hence, in c ~ h) we considered all 100 simulations for the comparison of the fraction of time points that multiple receptors are targeted simultaneously and the Gini coefficient of the frequency of targeted receptors. c, d) The low amino acid matching threshold for the receptor binding (ϵ seqM) facilitates viruses to target multiple receptors. e, f) The low survival rate of an offspring from both infected parents results in viruses targeting more specific receptors for more robust receptor binding. g, h) The high disease related death rate (λ D) causes more specialized receptor targeting. (PDF 473 kb) [file 12862_2016_804_MOESM5_ESM.pdf]

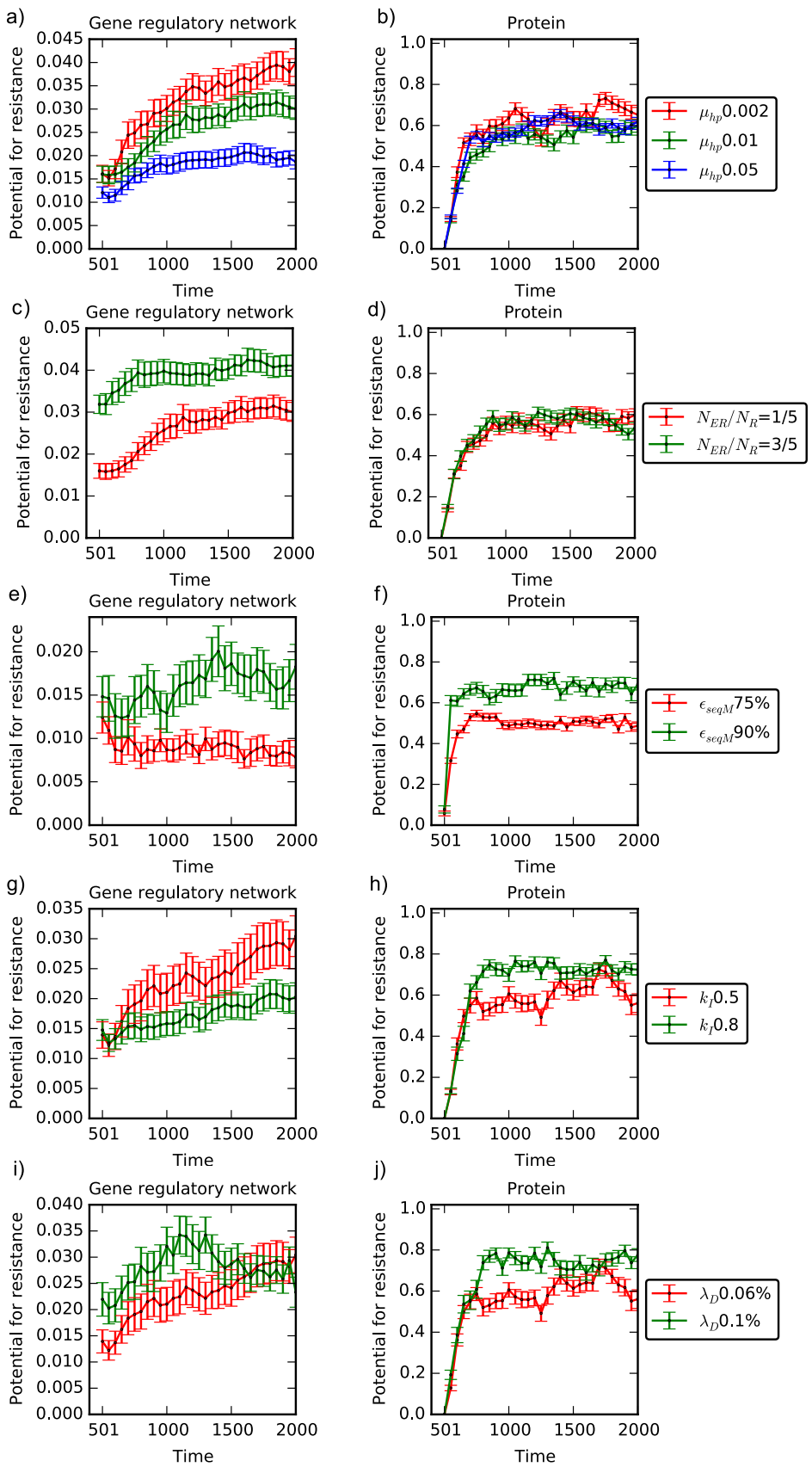

Supplement: Additional file 7: Figure S6. — Evolutionary potential for resistance in the gene regulatory network and receptor proteins for different conditions. For susceptible host population, the ability to resist using GRN rewiring (1st column) and protein binding site changes (2nd column) is measured for different a, b) host protein mutation rates (μ hp), c, d) number of required expressed receptors (N ER), e, f) amino acid matching threshold for the receptor binding (ϵ seqM), g, h) survival rate from both infected parents (k I) and i, j) disease related death rate (λ D) (Error bar: std. dev. over 100 simulations). For low ϵ seqM and k I, population dynamics generally follows that of Additional file 2: Figure S1 b. Hence, in e ~ h) we considered all 100 simulations for the comparison of the resistance potentials. a, b) For lower μ hp, hosts evolve a GRN based strategy (L = 30, μ hp = 0.01, ϵ seqM = 90 %, k I = 0.8). c, d) When expression of more receptors is required, hosts evolve the potential for resistance using GRN rewiring to higher level. (L = 30, N ER/N R = 3/5, ϵ seqM = 90 %, k I = 0.8), e, f) When receptor binding is simple (short L), for reduced ϵ seqM hosts does not necessarily evolve the potential for a GRN rewiring strategy (L = 10, μ hp = 0.002, N ER/N R = 3/5, k I = 0.8). g, h) Selection pressure triggered by the low k I evolves the potential for GRN rewiring strategy (L = 30, μ hp = 0.002, N ER/N R = 3/5, ϵ seqM = 90 %). i, j) The potential for resistance using network rewiring increases both for low and high diseases related death rates (λ D). (PDF 2128 kb) [file 12862_2016_804_MOESM7_ESM.pdf]

a)

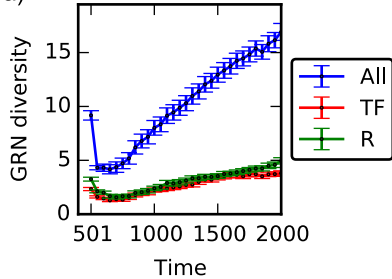

b)

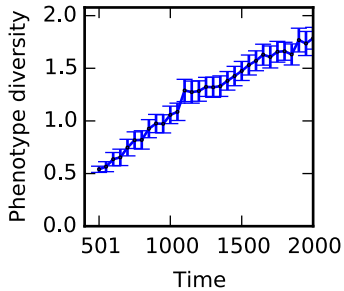

c)

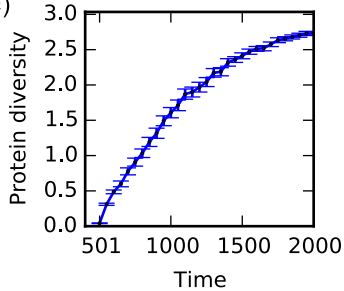

Supplement: Additional file 8: Figure S7. — Increased genetic diversity in the gene regulatory networks, phenotypes and receptor proteins. Genetic diversity is measured using the Margalef index (see the last section in Results). a) whole GRNs (blue), transcription factor regulation sub-networks (red), receptor regulation sub-networks (green) of susceptible hosts. b) Phenotypes (gene expression levels) of susceptible populations. c) Receptor sequence of susceptible populations. (PDF 461 kb) [file 12862_2016_804_MOESM8_ESM.pdf]

a)

Resistance using regulatory changes

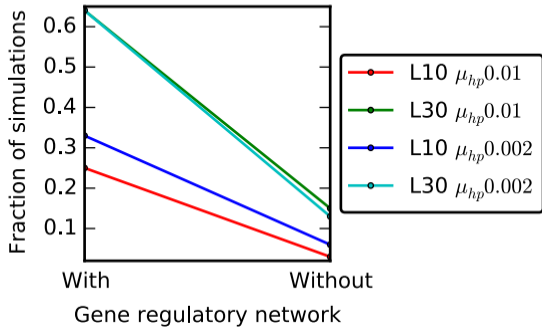

b)

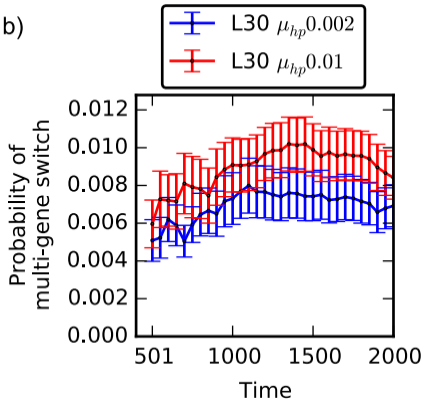

Supplement: Additional file 9: Figure S8. — The effect of having a complex gene regulatory network (GRN) for controlling receptor gene expression. a) Preference for resistance using GRN rewiring to protein mutations decreases when there are no regulatory interactions between genes (without regulatory interactions in the gene network) (N ER/N R = 3/5, ϵ seqM = 90 %, k I = 0.8). b) The ability to switch the expression of multiple receptors with a complex GRN. The probability of multiple receptor gene expression switching (see Methods) increases during host-virus coevolution (L = 30, μ hp = 0.01 and 0.002, ϵ seqM = 90 %, k I = 0.8). (PDF 245 kb) [file 12862_2016_804_MOESM9_ESM.pdf]
