# Supplementary material for: Potential for evolution of complex defense strategies in a multi-scale model of virus-host coevolution
Source: BMC Evol Biol. 2016 Oct 26;16:233. doi: 10.1186/s12862-016-0804-z (PMC5080737; doi:10.1186/s12862-016-0804-z)
Supplement: Additional file 6: Figure S5. — Preference for resistance using gene regulatory network (GRN) rewiring to protein mutations under different conditions. The fraction of simulations where GRN rewiring strategy is used more often than the protein binding site change strategy for resistance for different a) required number of expressed receptors (N ER), b) amino acid matching threshold for the receptor binding (ϵ seqM), c) survival rate from both infected parents (k I) and d) disease related death rate (λ D). For low ϵ seqM, k I and λ D, the population dynamics generally follows that shown in Additional file 2: Figure S1 b. Hence, in b, c, d) we considered all 100 simulations for the comparison of the preference for resistance using GRN rewiring to protein mutations. a) As more receptors are required to be expressed (higher N ER), hosts preferentially use GRN rewiring less often than protein mutations. b) When the binding complexity is low, for lower amino acid matching threshold for the receptor binding (ϵ seqM), hosts do not preferentially select GRN rewiring strategy. c) When k I is low, hosts does not favor the GRN rewiring strategy. d) When the disease related death rate (λ D) is high, hosts hosts less favor the GRN rewiring strategy for resistance. (PDF 208 kb) [file 12862_2016_804_MOESM6_ESM.pdf]

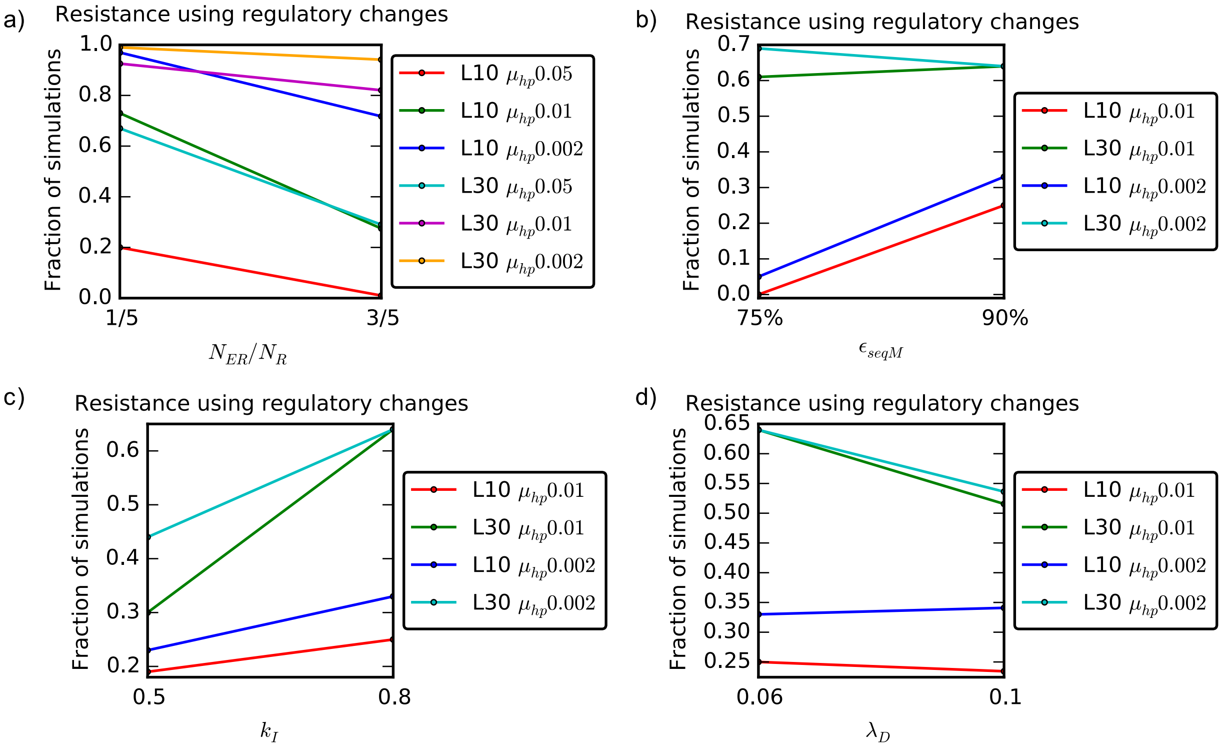
a)
Resistance using regulatory changes
Fraction of simulations
1.0
0.8
0.6
0.4
0.2
0.0
1/5
3/5
N_{ER}/N_R
L10 $\mu_{hp}0.05$
L10 $\mu_{hp}0.01$
L10 $\mu_{hp}0.002$
L30 $\mu_{hp}0.05$
L30 $\mu_{hp}0.01$
L30 $\mu_{hp}0.002$
b)
Resistance using regulatory changes
Fraction of simulations
0.7
0.6
0.5
0.4
0.3
0.2
0.1
0.0
75%
90%
ϵ_{seqM}
L10 $\mu_{hp}0.01$
L30 $\mu_{hp}0.01$
L10 $\mu_{hp}0.002$
L30 $\mu_{hp}0.002$
c)
Resistance using regulatory changes
Fraction of simulations
0.6
0.5
0.4
0.3
0.2
0.5
0.8
k_I
L10 $\mu_{hp}0.01$
L30 $\mu_{hp}0.01$
L10 $\mu_{hp}0.002$
L30 $\mu_{hp}0.002$
d)
Resistance using regulatory changes
Fraction of simulations
0.65
0.60
0.55
0.50
0.45
0.40
0.35
0.30
0.25
0.06
0.1
λ_D
L10 $\mu_{hp}0.01$
L30 $\mu_{hp}0.01$
L10 $\mu_{hp}0.002$
L30 $\mu_{hp}0.002$
